# Swallowing disorders and mortality in adults with advanced cancer outside the head and neck and upper gastrointestinal tract: a systematic review

**DOI:** 10.1186/s12904-023-01268-4

**Published:** 2023-10-06

**Authors:** Danielle Nunes Moura Silva, Laélia Cristina Caseiro Vicente, Vanessa Laís Pontes Glória, Amélia Augusta de Lima Friche

**Affiliations:** 1 Universidade Federal de Minas Gerais (UFMG), Pós-Graduação Em Ciências Fonoaudiológicas, Faculdade de Medicina, 190 Alfredo Balena Avenue, Santa Efigênia, Belo Horizonte City, Minas Gerais State 30130-100 Brazil; 2 Instituto de Previdência Dos Servidores Do Estado de Minas Gerais (IPSEMG), IPSEMG Hospital, 225 Alameda Ezequiel Dias, Centro, Belo Horizonte City, Minas Gerais State 30130-110 Brazil

**Keywords:** Deglutition disorders, Neoplasms, Prognosis, Survival, Palliative care, Systematic review

## Abstract

**Background:**

Although oncological palliative care is increasingly being offered by multidisciplinary teams, there is still a lack of data about some symptoms handled by these teams, such as dysphagia, in patients with advanced cancer outside swallow regions. This study aimed to estimate the occurrence of dysphagia in prognosis studies of adults with advanced cancer outside the head, neck, and upper gastrointestinal tract, and to determine if there is an association with mortality.

**Methods:**

A systematic review of studies that evaluated dysphagia and mortality was conducted (PROSPERO: CRD42021257172).

**Data sources:**

BVS, PubMed, CINAHL, Web of Science, and Scopus. Data between 2011 and 2023 were selected.

**Results:**

Among the 608 articles screened, only 14 were included, which covered different types of cancer, primarily Lung, and Genitourinary, Skin, Hematological, and Central Nervous System as well. Dysphagia demonstrated a variable frequency, and almost half of the studies found a percentage of dysphagia above 60%, appearing most as a symptom that affects health-related quality of life and prove to be a toxicity of treatment. The association between dysphagia and mortality was only evaluated in three articles that studied advanced lung cancer, in which, after controlling for covariates, swallowing disorders were associated with worse survival, with prevalences of dysphagia and hazard ratios of 78.5% (1.12 [1.04–1.20]), 4% (1.34 [1.28–1.35]), and 3% (1.40 [1.07–1.81]), respectively.

**Conclusions:**

The occurrence of dysphagia in advanced cancer outside the head, neck, and upper GI tract is common, and there seems to be an association with significantly decreased survival in patients with advanced lung cancer.

**Supplementary Information:**

The online version contains supplementary material available at 10.1186/s12904-023-01268-4.

## Background

Cancer is a leading cause of increased morbidity and mortality worldwide, accounting for nearly 10 million deaths in 2020, highlighting the need for studies on advanced cancer [1]. Consequently, there has been an increase in the number of people with dependence who often experience an ongoing deterioration in health-related quality of life (HRQoL) [2] and thus need palliative care. Previous studies and meta-analyses have demonstrated that symptoms that contribute to decreased HRQoL can influence survival across many cancer types, independent of sociodemographic and other clinical prognostic factors [2–7].

Current evidence suggests that access to specialist palliative care consisting of a multidisciplinary team is required to facilitate the management of patients with multiple care needs [8]. Patients with all types of advanced cancers and oncological treatments may develop different side-effects and often experience deterioration in functional status, all of which can affect their HRQoL [2]. One such side effect is dysphagia, which is a complex loss of swallowing function and is a multifactorial symptom that may be caused by a variety of reasons and mechanisms [9]. In oncology, dysphagia is commonly associated with head and neck or upper gastrointestinal (GI) tumors, although a previous review and a cross-sectional study revealed that swallowing difficulty also occurs in those with tumors outside anatomic swallow regions [10, 11].

A multidisciplinary team can track the patient’s worsening swallowing function and provide a stimulus to help the patient achieve better comfort, thus helping monitor this functional degradation [10, 12], which could assist the team in the general clinical prognosis. Furthermore, estimating and communicating prognosis based on functional capacity both before and during the illness is a crucial step in improving patient-centered clinical decision-making and ensuring that patients’ care matches their goals, values, and preferences [13]. However, more research is needed to elucidate survival concerning swallowing disorders among patients with advanced cancer outside the head, neck, and upper GI tract. Further research on this topic is necessary, particularly within prognostic studies involving patients in palliative care, to improve the monitoring of burden symptoms as potential prognostic factors. This, in turn, will facilitate the development of statements to guide decision-making concerning swallowing disorders in patients with advanced cancer outside the head, neck, and upper GI tract. This systematic review aimed to answer the following research questions: Is dysphagia a symptom frequently reported in prognostic studies of adults with advanced cancer outside the head, neck, and upper GI tract? Is there an association between survival and dysphagia in adults with advanced cancer outside of the head, neck, and upper GI tract?

## Methods

### Study design

This study aimed to explore if dysphagia is a frequently reported symptom in the prognostic studies of adults with advanced cancer outside the head, neck and upper GI tract. It also explored the possibility of an association between survival and dysphagia of these population. For this study, a systematic review was performed and reported in accordance with the Preferred Reporting Items for Systematic reviews and Meta-analyses (PRISMA) [14]. The protocol was registered with the International Prospective Register of Systematic Reviews (PROSPERO) under the ID CRD42021257172.

### Search strategy and selection criteria

The following electronic databases were searched: LILACS, BBO, IBECS (BVS^1^), MEDLINE/PubMed (NIH), CINAHL (EBSCO Host), SCOPUS (Elsevier), and Web of Science (Clarivate Analytics), using terminology related to “deglutition disorders” and “survival” and “palliative care,” while excluding terminology related to “head and neck neoplasms” or “esophageal neoplasms” or “stomach neoplasms.” Common synonyms for survival, palliative care, and neoplasms were included, and the combined terminology was specifically adjusted for each database. Only studies published in the last twelve years (between January 1, 2011, and August 31, 2023) were searched because this is the same period in which worldwide substantial growth of palliative care was observed, according to the quality of death index constructed by an Economist Intelligence Unit (EIU) [15], which consequently increased the number of publications in this area. The detailed search strategy for each database is included in the Supplementary Material section.

The eligibility criteria were confirmed through title, abstract, and full-text screenings. Observational and prognostic studies that analyzed dysphagia in patients with advanced cancer were included. Studies on adults with primary site cancer in the anatomic swallow regions were excluded. If a study did not specify the cancer diagnosis or mentioned a category of “other” (without a clear explanation of which types of cancer made up “other”), these studies were also excluded. The eligibility criteria are listed in Table 1.

**Table 1:** Eligibility criteria of the studies for the systematic review

Component criteria
**Study population**
*Inclusion criteria*
•Adults (⩾18 years old)
•Diagnosed with dysphagia
•Advanced cancer, with evidence of at least one of the following criteria:
(1) Metastatic primary solid cancer
(2) Locally advanced solid cancers
(3) Advanced hematological neoplasms
*Exclusion criteria*
•Primary site cancer in head and neck or upper gastrointestinal tract
**Study design**
*Inclusion criteria*
•Observational studies: retrospective or prospective studies
•Prognostic studies
*Exclusion criteria*
•Review articles, discussions, letters, editorials, comments, case reports, case series, case studies, cross-sectional studies, and qualitative studies
**Outcome**
Survival
Prevalence of deglutition disorders
**Languages**
English, Spanish, and Brazilian Portuguese
**Years**
Studies published from 2011 to 2023

The reference selection process was divided into four phases. In Phase 1, one reviewer (D.N.M.S.) excluded duplicates using the Rayyan QCRI platform [16]. In Phase 2, two reviewers (D.N.M.S. and V.L.P.G.) independently and blindly screened all titles and abstracts, excluding irrelevant papers and records that did not meet the inclusion criteria or met any other item described in the exclusion criteria (Table 1). This blind process was ensured and registered using the Rayyan QCRI platform [16]. Differences in decisions between the two authors were discussed until an agreement was reached.

In Phase 3, the same reviewers (D.N.M.S. and V.L.P.G.) applied the eligibility criteria to the full text of the studies selected in Phase 2. During this phase of reading the entire article, if the reviewers found studies involving patients with primary site cancer in the head and neck or upper gastrointestinal tract, or those with brain metastases, or if they met any other item described in the exclusion criteria that had not been excluded in Phase 2 (due to the abstract not indicating it), they were excluded. When necessary, a third reviewer (A.A.L.F.) was consulted to reach a consensus in cases of disagreement between the first two reviewers.

Finally, in Phase 4, the reviewers (D.N.M.S. and V.L.P.G.) checked the reference lists of the studies selected in Phase 3 and, once again, independently and blindly screened all titles and abstracts, as described in Phases 2 and 3. They excluded records and applied the eligibility criteria to the full text of the selected studies. Differences in decisions between the two reviewers were also resolved by the third reviewer (A.A.L.F.). A Cochrane review [17] discussed supplementary search techniques in systematic reviews, such as checking reference lists, and concluded that there is some evidence to support this method. This prompted us to include handsearching.

### Data extraction and synthesis

Data were extracted from all included studies (by D.N.M.S.) using a spreadsheet. The following information was recorded for each study: author/year, place of study, study design, study population (cancer types, sample size, number of men and women, and measures of central tendency of age), healthcare setting, objectives, prevalence of dysphagia, diagnostic evaluation of dysphagia, outcomes, survival, types of survival analyses used, survival and dysphagia association, other prognostic factors, main results, and conclusions.

### Quality assessment

The articles included in this review comprised a heterogeneous range of methods that required multiple assessment tools. For quality assessment, all studies were evaluated by applying the Strengthening the Reporting of Observational Studies in Epidemiology (STROBE) initiative [18] and the Quality In Prognosis Studies (QUIPS) tool [19] to determine methodological quality and risk of bias, respectively. The STROBE was chosen as a checklist for observational studies. The maximum score was 22 points, distributed as follows: title and/or summary (one item), introduction (two items), methodological aspects (nine items), results (five items), discussion (four items), and other information (one item on financing). For each study, one of the 22 items received a score ranging from 0 or 1 when considering whether it met or did not meet each criterion. Based on this, three categories were established for quality assessment: A, studies that met more than 80% of the criteria; B, studies that achieved 50% to 80% of the criteria; and C, studies that met less than 50% of the criteria. The QUIPS tool was chosen because it is the recommended one for prognostic factor studies by the Cochrane Prognosis Methods Group, which has six domains for evaluating validity and bias: study participation, study attrition, prognostic factor measurement, outcome measurement, study confounding, and lastly, statistical analysis and reporting. The risk of bias was expressed on a three-grade scale (high, moderate, or low).

The quality of evidence was assessed using the Grading of Recommendations Assessment, Development and Evaluation (GRADE) system [20], which uses a four-grade scale: A) high evidence, B) moderate evidence, C) low evidence, and D) very low evidence.

No studies were excluded based on the quality assessment.

## Results

**Fig. 1:**
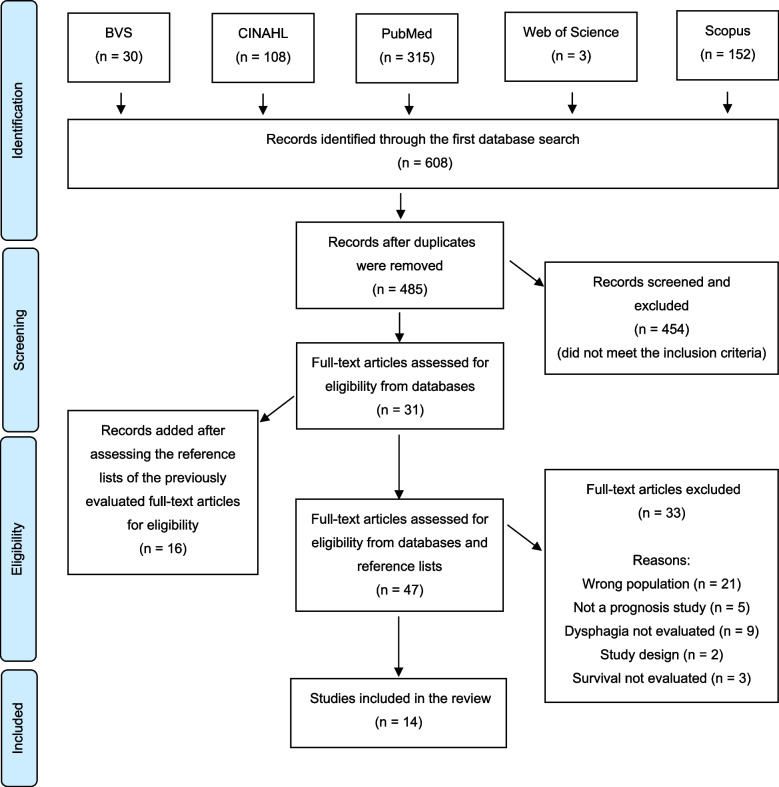
PRISMA flow diagram

A total of 608 records were identified using the search criteria. After removing duplicates, 485 articles were screened through their abstracts. Thirty-one articles were selected for full-text review, and after assessing their reference lists, 16 additional articles were included. Ultimately, 14 articles were chosen for final inclusion. Figure 1 provides a detailed depiction of this process, utilizing a flow diagram illustrating the literature search and selection criteria adapted from PRISMA.

Six studies reported data from the last five years [21–26]. Three studies were multicenter [2, 27, 28], for which the authors conducted research in European countries, and one [28] in North America countries. Another study was conducted across two continents simultaneously (North America and Europe) [29]. The rest of the studies were only conducted in one country on the following continents: one in North America [25], one in Europe [30], three in Asia [24, 26, 31], and one in Australia [22]. Nine studies were cohort studies [21–26, 31–33], whereas the others were randomized trials [2, 28–30]. Table 2 provides an overview of the studies included for this systematic review.

**Table 2:** Summary of the included articles

**1º Author, Year**	**Type of cancer**	**Participants (N/Sex/Age)**	**Prevalence of dysphagi**a** (N/%)**	**Diagnostic evaluation of dysphagia (How/Which)**	**Survival (mean or median/HR[CI]/rate)**	**Survival and dysphagia association (days/HR[CI])**
Liu et al., 2023 [26]	Lung cancer (NSCLC and SCLC)	3634 (F = 1209, M = 2425); Mean age (SD): 60.2y (± 9.86)	3%	Frequency during the hospital stay and 30 days after hospital admission (telephone follow-up): Patient-Generated Subjective Global Assessment*	Median = Non-NIS group: 20.6 months, NIS group: 31.2 months, *p* ≤ 0.001	NIA days / 1.401 [1.07–1.81]
Marmor et al., 2020 [25]	Lung cancer (NSCLC and SCLC)	201.674 (F = 95.144, M = 106.530) Age: < 80y: 82% of N total	8517 (4%)	Retrospective data: ICD-9-CM (diagnostic codes for dysphagia) and CPT	Median = with dysphagia: 8 months [95%CI 7–9] HR: 1.34 [95%CI 1.28–1.35] without dysphagia: 12 months [95%CI 11–13], *p* ≤ 0.0001	NIA days / 1.34 [1.28–1.35]
Abbas et al., 2019 [22]	Lung cancer (NSCLC)	63 (F = 22, M = 41) Median age (interquartile range) = 66.6y (57.2–72.1)	4 (7.1%) of 56 patients alive after 6 months	Frequency not reported: CTCAE version 3.0*	Median = 21 months	Not
Markos et al., 2019 [21]	Lung cancer (NSCLC)	42 (F = 7, M = 35) Mean age (range) = 68.4y (52–80)	All patients	Frequency not reported: cites the stages, but does not refer to which protocol	Mean = 4 months	Not
Arscott et al., 2018 [23]	Melanoma Lung cancer (NSCLC) Kidney cancer Liver cancer Myeloma Sarcoma	30 (F = 9, M = 21) Median age at time of RT (range) = 61y (27–82)	9 (30%)	Frequency not reported: CTCAE version 4.0*	Median = 87 days	Not
Kim et al., 2018 [24]	Lung cancer (NSCLC and SCLC)	84 (F = 23, M = 61) Mean age (SD) = Stent group: 62.4y (± 11.5), Gastrostomy group: 58.5y (± 6.3)	All patients	Retrospective data: SEMS or PG placement	HR: 0.682 *p* = 0.219 (between SEMS and PG)	Not
Hatton et al., 2016 [30]	Lung cancer (NSCLC)	18 (F = 4, M = 14) Mean age (range) 70y (44–84)	16%	Frequency not reported: CTCAE version 4.0*	Rate: 2 years of survival = 49%	Not
Thier et al., 2016 [32]	Glioblastoma	57 (F = 18, M = 39) Mean age at death (SD) = 59y (± 11)	37 (65%)	Daily: Signs, symptoms, and treatment strategies were registered using the standardized protocol + additional information from the patients’ charts*	Mean = 48 weeks	Not
Bradley et al., 2015 [28]	Lung cancer (NSCLC)	465 (F = 188, M = 277) Median (range) = 60 Gy group: 64y (38–83), 74 Gy group: 64y (41–83), Cetuximab group: 64y (38–83), No-cetuximab group: 64y (37–82)	139 (31%)	Frequency not reported: CTCAE version 3.0*	Median (months) 60 Gy group: 28.7 = 1.38 [1·09–1·76], 74 Gy group: 20.3, p = 0.004 | Cetuximab group: 25 = 1.07 [0.84–1.35], No-cetuximab group: 24.0, *p* = 0,29	Not
Koekkoek et al., 2014 [27]	Highgrade Glioma	178 (F = 53, M = 125); Mean age at diagnosis, years (SD)—59.7 (12.5)	Dysphagia prevalence at 3 months before death = 7.5%, Dysphagia prevalence at 1 week before death = 24.5%	Physicians who had been involved in EOL care for these patients were invited to complete questionnaires on the EOL phase	Median = Group with grade IV—10.6 months [9.2–12.1], Group with grade III = 12.4 months [10.6–14.1], p ≥ .05	Not
Ansari et al., 2014 [31]	Thymoma	45 (F = 18, M = 27) Mean age (range) = 43y (45.4 ± 17.7)	3 (7%)	Every 3 months for the first 2 years Every 4 months during the third year Every 6 months in the fourth and fifth years and annually thereafter: history and physical examination*	Rate (5 years of survival = 70.8%, 10 years of survival = 62.9%)	Not
Ediebah et al., 2014 [2]	Lung cancer (NSCLC)	391 (F = 136, M = 255) Median age (range) = Group A: 57y (27–75), Group B: 57y (28–75), Group C: 56y (31–75)	307 (78.5%)	Every 6 weeks: global health status/QOL scale*	Median = (F = 9.6 months, M = 7.2 months)	NIA days / 1.12 [1.04–1.20]
Oberije et al., 2014 [33]	Lung cancer (NSCLC and SCLC)	155 (F = 69, M = 86) Mean age (SD) = NSCLC group: 64.7y (± 10.5), SCLC group: 65.5y (± 8.8)	Grade ≥ 3 dysphagia = NSCLC group: 14 (11.6%), SCLC group: 3 (12.0%)	Any timepoint during or after the end of RT, with a maximum of 4 weeks: CTCAE version 3.0*	NIA	Not
Schuette et al., 2012 [29]	Lung cancer (NSCLC)	95 (F = 29, M = 66) Mean age (SD) = Placebo group: 64.2y (± 7.7), Treatment group: 61.6y (± 9.8)	Grade ≥ 2 dysphagia = Palifermin group: 30 subjects (61%), Placebo group: 32 subjects (70%) Grade ≥ 3 dysphagia = Palifermin group: 11 subjects (22%), Placebo group: 13 subjects (28%) Grade 4 dysphagia = reported for one subject in the palifermin group	Twice weekly: CTCAE version 3.0*	Median = Placebo: 319 days, Palifermin: 513 days	Not

*N* total sample, *HR h*azard ratio, *CI *confidence interval, *F *female, *M *male, *SD *standard deviation, *y *years old, *RT *radiotherapy, *NSCLC *non-small cell lung cancer, *SCLC *small cell lung cancer, *not exclusively to evaluate dysphagia, *NIS *nutrition impact symptoms, *EOL* end-of-life, *CTCAE *NCI common terminology criteria for adverse events, *SEMS *self-expandable metallic stent, *PG* percutaneous gastrostomy, *NIA* no information available, *ICD-9-CM* International Classification of Diseases, Ninth Revision, Clinical Modification, *CPT* Current Procedural Terminology

Seven articles did not report the settings of the studies [2, 21, 23–25, 30, 33] but when cited, most were performed in hospitals. Three studies [26, 31, 32] were completed in and used data exclusively from hospital healthcare settings, while the other four [22, 27–29] were conducted in two healthcare settings simultaneously (outpatient, hospital, or homecare).

Seven articles reported deterioration in functional status of the patients; two found good performance status in the upper 40% of the overall sample using the World Health Organization performance status (WHO-PS) [30, 33]. Another study used the Eastern Cooperative Oncology Group Performance Status (ECOG-PS) [29], which demonstrated 96% functional independence of the sample. Two studies showed severely disabled functionality using the Karnofsky Performance Status Scale (KPS) [27, 32].

Ten studies were conducted on a population with advanced lung cancer [2, 21, 22, 24–26, 28–30, 33]. One study was composed of a population that had been diagnosed with six types of cancer: sarcoma, myeloma, melanoma, as well as lung, kidney, and liver cancer, without making a comparison between them [23]. One article analyzed the glioblastoma population [32], one Highgrade Glioma [27], and another investigated patients with thymoma [31]. Most studies reported survival data in the mean or median format by day, week, or month. The lower overall survival median was 87 days in a retrospective cohort [23] and the lower overall survival mean was 48 weeks in a prospective study [32].

**Fig. 2:**
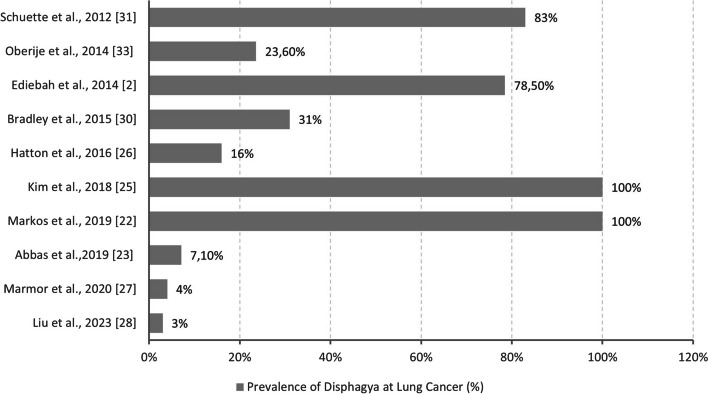
Prevalence of dysphagia at subgroup lung cancer

A prevalence of dysphagia above 60% has been reported in five the studies considered in this review [2, 21, 24, 29, 32]. In the remaining articles, the figure varied between 3 and 30% [22, 23, 25–28, 30, 31, 33]. Nine [2, 21–23, 28–32] studies related the possible cause of dysphagia to toxicity of cancer treatment, and only three articles found dysphagia to be a symptom of cancer itself [21, 24, 27]. Furthermore, only one article focused on patients with diagnoses of dysphagia prior to their cancer diagnoses [25]. Only two articles [21, 24] specified the type of dysphagia and described it as esophageal dysphagia. No studies have been designed exclusively to evaluate dysphagia in cancer outside the head, neck, and upper GI tract. Nevertheless, because many of the studies are in lung cancer, we highlight the prevalence of dysphagia in this subgroup in Fig. 2.

Only one multicenter randomized trial [2] and two cohort [25, 26] measured the impact of dysphagia upon survival and found that dysphagia increased the risk of death with hazard ratios (HRs) of 1.12, 1.34 and 1.40, respectively. All these HRs were adjusted for clinical and socio-demographic covariates, as like including age, sex, race, urban/rural status of treatment facility, functional status, histological subtype, and clinical stage.

### Quality of studies

Three studies had a low risk of bias associated with Level A of methodological assessment. Most studies presented high and moderate risks of bias, often due to the observed effect of the prognostic factors on the outcome, which was very likely to be distorted by confounders and because the selected statistical model produced spurious or biased results.

The evaluation of the quality of evidence by GRADE was applied to the 14 articles, graded as follows: four were Level A [2, 24, 26, 31], five were Level B [23, 25, 28–30], five were Level C [21, 22, 27, 32, 33], and none were Level D. Of the three articles that investigated the relationship between survival and dysphagia, two were graded at Level A [2, 26] and the other at level B [25].

The details of the quality assessments (methodology and evidence) are provided in Table 3.

**Table 3:** Quality assessmen﻿t

References and year of publication	STROBE	QUIPS	GRADE
*Liu *et al*., 2023 *[26]	20 A Level	HIGH 1-M; 2-L; 3-H; 4-H; 5-H; 6-M	A
*Marmor S, 2020 *[25]	20 A Level	MODERATE 1-L; 2-M; 3-M; 4-L; 5-L; 6-L	B
*Abbas MN *et al*., 2019 *[22]	20 A Level	HIGH 1-M; 2-L; 3-L; 4-L; 5-H; 6-M	C
*Markos P *et al*., 2019 *[21]	11 B Level	HIGH 1-H; 2-H; 3-M; 4-H; 5-H; 6-H	C
*Arscott W *et al*., 2018 *[23]	19 A Level	HIGH 1-L; 2-L; 3-L; 4-M; 5-H; 6-H	B
*Kim J *et al*., 2018 *[24]	19 A Level	LOW 1-M; 2-L; 3-L; 4-L; 5-L; 6-L	A
*Hatton MQ *et al*., 2016 *[30]	14 B Level	HIGH 1-H; 2-M; 3-M; 4-M; 5-H; 6-H	B
*Thier K *et al*., 2016 *[32]	18 A Level	HIGH 1-L; 2-M; 3-L; 4-L; 5-H; 6-H	C
*Bradley *et al*., 2015 *[28]	15 B Level	HIGH 1-H; 2-H; 3-M; 4-M; 5-H; 6-H	B
*Koekkoek *et al*., 2014 *[27]	17 A Level	LOW 1-M; 2-L; 3-L; 4-L; 5-H; 6-L	C
*Ansari M *et al*., 2014 *[31]	17 A Level	LOW 1-L; 2-L; 3-L; 4-L; 5-L; 6-L	A
*Ediebah DE *et al*., 2014 *[2]	20 A Level	LOW 1-L; 2-L; 3-L; 4-M; 5-L; 6-L	A
*Oberije C *et al*., 2014 *[33]	17 A Level	HIGH 1-L; 2-M; 3-L; 4-L; 5-H; 6-M	C
*Schuette W *et al*., 2012 *[29]	20 A Level	MODERATE 1-L; 2-L; 3-M; 4-M; 5-M; 6-L	B

*STROBE* Studies were scored by quality using Strengthening the Reporting of Observational Studies in Epidemiology initiative: A Level = more than 80% (≥ 17), B Level = 50 to 80% (10 until 16)

*QUIPS* Quality In Prognosis Studies tool**:** 1 – Study participation, 2—Study attrition, 3—Prognostic factor measurement, 4—Outcome measurement, 5—Study confounding, 6—Statistical analysis and reporting, *L* Low risk of bias, *M* Moderate risk of bias, *H* High risk of bias, *QUIPS LOW* 6 low´s or 5 low's + 1 moderate, *QUIPS MODERATE* 6 moderate's or 2 moderate´s + 4 low's, *QUIPS HIGH* 6 high's or ≥ 1high´s or ≥ 3 moderate's

*GRADE* Grading of Recommendations Assessment, Development and Evaluation system: A = High evidence, B = Moderate evidence, C = Low evidence

## Discussion

This systematic review of prognosis studies involving patients with advanced cancer outside the head, neck, and upper GI tract revealed that the occurrence rate of dysphagia ranged from 4 to 78%, with an association with survival represented by hazard ratios ranging between 1.12 and 1.40. These high levels of variability likely stem from differences in demographics, sample sizes, cancer types, oncological treatments, types of dysphagia, measurement tools for assessing swallowing disorders, clinical assessment frequency, and healthcare settings. This combination may limit our ability to firmly establish the true prevalence of dysphagia and its association with survival in this specific population. Nevertheless, when examined in detail, the articles addressed at least one of the two research questions, prompting us to document the issues preventing the answering of all research questions and the possibility of conducting a meta-analysis. Furthermore, it is necessary to discuss swallowing disorders in these types of tumors outside of anatomical swallowing regions precisely because the decision-making process regarding nutrition and hydration in the care of this population remains unclear. Therefore, these issues need to be explored and carefully analyzed.

It is important to emphasize that the decision-making process regarding nutrition and hydration in the care of a patient with advanced head and neck cancer is generally complex [34]. However, in these cases, there is already literature favoring the use of an alternative route as early as possible, based on the well-known high prevalence of dysphagia (> 60%) and its close association with increased physical suffering [35, 36].

Our review highlights the disparity in dysphagia occurrence in patients with advanced cancer outside the head, neck, and upper GI tract. There is little evidence that people have dysphagia, even though they are already known to suffer from distressing GI symptoms. This may, in part, be related to the absence of studies designed exclusively to evaluate dysphagia in cancer outside the head, neck, and upper GI tract. Moreover, no information was available about the use of tools designed exclusively for dysphagia screening and evaluation in either of the methodological designs of articles selected at out review, except for the study on pre-existing dysphagia [25], which focused on specific dysphagia aspects. The lack of specific tools may affect the ability to distinguish dysphagia from other gastrointestinal disorders. A previous systematic review also demonstrated the lack of tools for exclusively examining dysphagia as a gap in the existing literature [10].

Only one study showed an association between dysphagia and functional impairment, with dysphagia being one of the main symptoms observed during the last 10 days before death [32]. Studies that take into account the functional status of the cancer patient population are still limited, underscoring a gap in the literature. Despite functionality rating scales having been publicly available for many years, most of the articles selected in our review did not utilize them or failed to elucidate the functional characteristics identified. Assessing functional impairment is essential to enhance our understanding of patient needs, their ability to perform daily activities, and their tolerance for therapies, particularly in the context of cancer [37].

The setting in which the study is conducted is one example that contributes to describing the functional status of the population. In outpatient settings, patients are accessed much earlier in the disease continuum and typically exhibit a better functional status. Conversely, in inpatient care, patients tend to be more unstable and are more likely to experience disruptions in various bodily functions, such as swallowing. However, in our review, most of the articles did not report the settings of the studies. Furthermore, knowing the patient’s functional impairment helps the multidisciplinary team to assess prognosis and the assessment of symptoms associated with this deterioration in functional status, such as dysphagia. Nevertheless, we did not find any associations in our review. This lack of data impacts prognostication and consequentially could make it more difficult for clinical decisions [13]. Thier et al. [32] were the only ones to possibly demonstrate an association between dysphagia and functional status because dysphagia is one of the most frequent symptoms in the last 10 days before death, corroborating a previous systematic review [10], which also revealed swallowing problems more frequently in the last week before death.

The potential causes of dysphagia in advanced malignant neoplasms outside the head, neck, and upper GI tract remain unclear in the existing literature. In our review, we identified possible associations that may broadly contribute to swallowing difficulties. It was observed that lung cancer, often associated with the toxicity of treatment, was the most common cause of swallowing problems [21, 22, 24, 26, 28, 29, 33]. This is likely due to its close relationship with the pathophysiology of swallowing. The studies included in our review encompassed different types of cancer, potentially influencing various mechanisms responsible for the development of dysphagia. Advanced cancers of different types outside the head, neck, and upper GI tract can disrupt swallowing due to its cancer nature and pathophysiology. This can include neurological or neuromuscular issues, esophageal or vagus nerve compression, and other causes related to the specific cancer type itself or the anti-cancer treatments [11].

However, despite the consideration of these listed potential causes, our review did not identify any studies that comprehensively explain the pathophysiology of deglutition disorders in individuals with advanced malignant tumors outside the head, neck, and upper GI tract, which concursed with the findings by Kenny et al. [10].

Ediebah et al. [2] elucidated the importance of frequently assessing symptoms. This is important first because these symptoms are common acute or late toxicities from cancer treatment and second because of their association with survival across different cancer types. In our systematic review, dysphagia was the most common short-term complication of chemoradiotherapy and/or RT. Moreover, Marmor et al. [25] clarified the importance of investigating the impact of dysphagia in patients with cancer. Their findings suggested that in patients with lung cancer those with previous dysphagia (from before they were diagnosed with cancer) have a significantly decreased survival rate. In other words, this not only feeds doubt about whether this relationship is due to cause or effect, but also shows an inversely proportional relationship with survival in people with cancer, which is already an important information.

We chose to research only studies that evaluated survival and prognosis because these types of studies mostly have the general objective of helping to make decisions about healthcare based on reliable prognostic information [38], especially for patients with chronic disease or without a cure, which is our case. Moreover, all healthcare professionals should become familiar with prognostic factors because they are an essential step in the decision-making process (DMP), as proposed by Forte et al. [39]. The DMP framework consists of four complementary steps. Step 1 focuses on the disease and probabilistic estimation of the prognosis. Step 2 focuses on the person, and emphasis is given to learning and active listening about patient values. Step 3 focuses on effective teamwork, contextualizing and linking diseases rates and probabilities to all known patient’s values, presenting a summary of which treatments the team considers as acceptable, recommended, potentially inappropriate, and futile. Finally, Step 4 focuses on seeking shared goals of care for the best and worst scenarios, ensuring that the patient’s values are respected, as well as a scientifically acceptable medical practice will be provided. Therefore, we focus on prognostic studies because these types of studies contribute to the DMP, a pivotal premise to be followed during the care of a seriously ill patient, aligning prognosis factors, Evidence-Based Practice (EBP), and Person-Centered Care (PCC), always being based on bioethical referents.

The risk of death associated with dysphagia in our review was comparable to that reported by Kenny et al. [10], which ensures that a question about swallowing function becomes a guideline and part of routine care, followed by validated tools to identify and manage dysphagia in a timely manner. In addition, this highlights the need for a multidisciplinary team to treat advanced cancer in terms of familiarity with the signs of dysphagia, especially for the palliative care team, which determines targeted interventions to reduce burden. Moreover, this review reveals an unmet need for palliative speech-language therapy for people with advanced cancer outside the head, neck, and upper GI tract, based on the potentially positive impact on cancer prognosis and improvement in the quality of life of these populations when undergoing specialized multidisciplinary palliative team monitoring.

### Strengths and weaknesses

This review has several limitations. First, this systematic review found that most observational studies did not control for all potential confounders, and not all included studies had comparison groups, as reflected by the heterogeneity of the findings, as evidenced by the different types of cancer sites, cancer treatments, dysphagia types, and other differences. However, we conducted an extensive quality assessment to allow readers to draw their own conclusions. Second, the heterogeneity among all quantitative and statistical analyses of survival might have introduced some bias in the interpretation of the analyses. Third, owing to the limited number of articles addressing symptom prevalence and the heterogeneity of the populations, meta-analysis could not be performed. This is further evidenced by the lack of data on dysphagia parameters according to cancer site and/or treatment type. Finally, it is possible that some studies were not identified due to the choice of search terms and the selection of databases. The broad term “swallow” was deliberately avoided in the search strategy. It was deemed unlikely that any studies of advanced cancer outside the head, neck, and GI tract would contain this term in their title or abstract. Nevertheless, it is possible that some dysphagia screening tools were missed because of the exclusion of these broad search terms. Together, these factors could limit the quality of the evidence collected and analyzed in this review. An alternative methodology such as a scoping review may be useful in the future to describe the dysphagia prevalence in this heterogeneous group.

Despite these limitations, one crucial strength is that this review provides the first systematic description of dysphagia in patients with cancer outside the head, neck, and GI tract in prognostic studies. These findings reinforce the importance of evaluating, monitoring, and treating dysphagia in cancer patients to help them achieve a better quality of life.

### What this study adds

This review demonstrates a lack of data about the periods of occurrence involving a swallowing disorder and the dysphagia pathophysiology of patients with advanced cancer outside the head, neck, and upper GI tract; however, it was illustrated that swallowing disorders are a common symptom burden, which seems to be associated with survival in such patients. Therefore, it is expected that these populations may take advantage of speech therapists’ palliative approach. More studies investigating survival and dysphagia association should be encouraged so that the decision-making for nutrition and hydration can be based on evidence.

Furthermore, these patients still have an unmet need for palliative care, especially to control symptoms such as dysphagia. Patients’ care, especially through a multidisciplinary approach, can provide effective and timely interventions. This may improve patients’ HRQoL and well-being.

## Conclusions

The occurrence of dysphagia in advanced cancers outside the head, neck, and upper GI tract is reported to be common, and there seems to be an association with significantly decreased survival in patients with advanced lung cancer. The prevalence of swallowing problems and their association with survival is still not well understood due to a lack of research using specific tools to swallowing evaluation.

### Supplementary Information

**Additional file 1.**

## Data Availability

All data generated or analysed during this study are included in this published article [and its supplementary information files].

## Abbreviations

BBO: *Base de dados Bibliografia Brasileira de Odontologia*
BVS: *Biblioteca Virtual em Saúde*
CI: Confidence interval
CINAHL: Cumulative Index to Nursing and Allied Health Literature
CTCAE: Common Terminology Criteria for Adverse Events
DMP: Decision-Making Process
EBP: Evidence-Based Practice
ECOG-PS: Eastern Cooperative Oncology Group Performance Status
EIU: Economist Intelligence Unit
EOL: End-of-life
F: Female
GI: Gastrointestinal
GRADE: Grading of Recommendations Assessment, Development and Evaluation
HR: Hazard ratio
HRQoL: Health-Related Quality of Life
IBECS: *Índice Bibliográfico Español en Ciencias de la Salud*
ID: Identity Document
KPS: Karnofsky Performance Status Scale
LILACS: *Índice da Literatura Científica e Técnica da América Latina e Caribe*
M: Male
MEDLINE: Medical Literature Analysis and Retrieval System Online
N: Total sample
NIA: No information available
NIH: National Institutes of Health
NIS: Nutritional Impact Symptoms
NSCLC: Non-Small Cell Lung Cancer
PCC: Person-Centered Care
PG: Percutaneous Gastrostomy
PRISMA: Preferred Reporting Items for Systematic reviews and Meta-analyses
PROSPERO: Prospective Register of Systematic Reviews
QUIPS: Quality In Prognosis Studies tool
RT: Radiotherapy
SCLC: Small Cell Lung Cancer
SD: Standard Deviation
SEMS: Self-Expandable Metallic Stent
STROBE: Strengthening the Reporting of Observational Studies in Epidemiology
WHO-PS: World Health Organization performance status

## Footnotes

1 The BVS (Virtual Health Library) is a Brazilian database that operates in collaboration with the network of cooperating centers in Latin America and the Caribbean.
