# Supplementary material for: Swallowing disorders and mortality in adults with advanced cancer outside the head and neck and upper gastrointestinal tract: a systematic review
Source: BMC Palliat Care. 2023 Oct 6;22:150. doi: 10.1186/s12904-023-01268-4 (PMC10557219; doi:10.1186/s12904-023-01268-4)
Supplement: Supplementary file 1 — Additional file 1. [file 12904_2023_1268_MOESM1_ESM.docx]

**SEARCH STRATEGY of Swallowing Disorders and Mortality in Adults with Advanced Cancer Outside the Head and Neck and Upper Gastrointestinal Tract: A Systematic Review**

Silva DNM; Vicente LCC; Glória VLP; Friche AAL

| **Search Strategy for Medline via PubMed** |  |
| --- | --- |
| "Deglutition Disorders"[All Fields] AND ("Palliative Care"[All Fields] OR "Hospice Care"[All Fields] OR "Terminal Care"[All Fields] OR "Medical Oncology"[All Fields] OR ("neoplasms"[MeSH Terms] OR "neoplasms"[All Fields])) AND (("mortality"[Subheading] OR "mortality"[All Fields] OR "survival"[All Fields] OR "survival"[MeSH Terms] OR "prognosis"[All Fields] OR "prognosis"[MeSH Terms]) OR "Survival Analysis"[All Fields] OR "Survival Rate"[All Fields] OR "Health Status"[All Fields]) NOT ("Head and Neck Neoplasms"[All Fields] OR "Esophageal Neoplasms"[All Fields] OR "Stomach Neoplasms"[All Fields]) | |

| **Search Strategy for LILACS, BBO, IBECS via BVS** |  |
| --- | --- |
| ("Deglutition Disorders" OR "Trastornos de Deglución" OR "Transtornos de Deglutição" OR Disfagia OR "Transtornos da Deglutição") AND ("Palliative Care" OR "Cuidados Paliativos" OR "Cuidados Paliativos" OR "Assistência Paliativa" OR "Cuidado Paliativo" OR "Cuidado Paliativo de Apoio" OR "Tratamento Paliativo" OR "Hospice Care" OR "Cuidados Paliativos al Final de la Vida" OR "Cuidados Paliativos na Terminalidade da Vida" OR "Cuidado Paliativo a Doentes Terminais" OR "Terminal Care" OR "Assistência Terminal" OR "Cuidado Terminal" OR "End of life" OR "Medical Oncology" OR "Oncología Médica" OR Oncologia OR Cancerologia OR "Oncologia Clínica" OR Neoplasms OR Neoplasias OR Câncer OR Neoplasia OR "Neoplasia Maligna" OR "Neoplasias Malignas" OR Neoplasmas OR Tumor OR "Tumor Maligno" OR Tumores OR "Tumores Malignos") AND (Survival OR Sobrevida OR Sobrevida OR "Sobrevivência (Saúde Pública)" OR "Survival Analysis" OR "Análisis de Supervivencia" OR "Análise de Sobrevida" OR "Análise de Sobrevivência" OR "Método Kaplan-Meier" OR "Survival Rate" OR "Tasa de Supervivencia" OR "Taxa de Sobrevida" OR "Taxa de Sobrevida Cumulativa" OR "Taxa de Sobrevivência" OR "Tempo de Sobrevida Médio" OR "Health Status" OR "Estado de Salud" OR "Nível de Saúde" OR "Condições de Saúde" OR Prognosis OR Prognóstico OR Prognóstico) AND NOT ("Head and Neck Neoplasms" OR "Neoplasias de Cabeza y Cuello" OR "Neoplasias de Cabeça e Pescoço" OR "Câncer da Cabeça" OR "Câncer de Cabeça e Pescoço" OR "Câncer do Pescoço" OR "Neoplasias do Pescoço" OR "Neoplasias do Trato Aerodigestório Superior" OR "Esophageal Neoplasms" OR "Neoplasias Esofágicas" OR "Neoplasias Esofágicas" OR "Câncer Esofágico" OR "Câncer de Esôfago" OR "Câncer do Esôfago" OR "Stomach Neoplasms" OR "Neoplasias Gastricas" OR "Neolpasias Gástricas" OR "Câncer Estomacal" OR "Câncer Gástrico" OR "Câncer de Estômago" OR "Câncer do Estômago" OR "Neoplasias do Estômago") | |

| **Search Strategy for CINAHL via EBSCO Host, SCOPUS via Elsevier, and Web of Science via Clarivate Analytics** |  |
| --- | --- |
| ("Deglutition Disorders") AND ("Palliative Care" OR "Hospice Care" OR "Terminal Care" OR "Medical Oncology" OR Neoplasms) AND (Survival OR "Survival Analysis" OR "Survival Rate" OR "Health Status" OR Prognosis) AND NOT ("Head and Neck Neoplasms" OR "Esophageal Neoplasms" OR "Stomach Neoplasms") | |
